# Effect of reduced energy density of close-up diets on dry matter intake, lactation performance and energy balance in multiparous Holstein cows

**DOI:** 10.1186/2049-1891-5-30

**Published:** 2014-05-29

**Authors:** Wenming Huang, Yujia Tian, Yajing Wang, Aminamu Simayi, Amingguli Yasheng, Zhaohai Wu, Shengli Li, Zhijun Cao

**Affiliations:** 1 State Key Laboratory of Animal Nutrition, College of Animal Science and Technology, China Agricultural University, Beijing 100193, China

**Keywords:** Dietary energy density, Dry matter intake, Energy balance, Lactation performance, Transition cow

## Abstract

Energy intake prepartum is critically important to health, milk performance, and profitability of dairy cows. The objective of this study was to determine the effect of reduced energy density of close-up diets on dry matter intake (DMI), lactation performance and energy balance (EB) in multiparous Holstein cows which were housed in a free-stall barn and fed for ad libitum intake. Thirty-nine dry cows were blocked and assigned randomly to three groups fed a high energy density diet [HD, n = 13; 6.8 MJ of net energy for lactation (NE_L_)/kg; 14.0% crude protein (CP) ], or a middle energy density diet (MD, n = 13; 6.2 MJ NE_L_/kg; 14.0% CP), or a low energy density diet (LD, n = 13; 5.4 MJ NE_L_/kg; 14.0% CP) from d 21 before expected day of calving. After parturition, all cows were fed the same lactation diet to d 70 in milk (DIM). The DMI and NE_L_ intake prepartum were decreased by the reduced energy density diets (*P* < 0.05). The LD group consumed 1.3 kg/d (DM) more diet compared with HD group in the last 24 h before calving. The milk yield and the postpartum DMI were increased by the reduced energy density diet prepartum (*P* < 0.05). The changes in BCS and BW prepartum and postpartum were not affected by prepartum diets. HD group had higher milk fat content and lower lactose content compared with LD group during the first 3 wk of lactation (*P* < 0.05). The energy consumption for HD, MD and LD groups were 149.8%, 126.2% and 101.1% of their calculated energy requirements prepartum (*P* < 0.05), and 72.7%, 73.1% and 75.2% during the first 4 wk postpartum, respectively. In conclusion, the low energy density prepartum diet was effective in controlling NE_L_ intake prepartum, and was beneficial in increasing DMI and milk yield, and alleviating negative EB postpartum.

## Introduction

The transition period is the most challenging time in the production cycle of dairy cows because of depressed dry matter intake (DMI) and marked changes in metabolism that support late gestation, parturition and the onset of milk synthesis. The abrupt increase in energy demands after parturition results in a negative energy balance (NEB) which can be met by mobilization of body fat reserves and by a decrease in insulin-dependent glucose utilization in non-mammary tissues [1]. Extensive mobilization of body fat reserves is associated with metabolic disorders such as fatty liver and ketosis. DMI rather than milk yield is the major driver of NEB [2]. Therefore, nutritional management during the transition period designed to increase postpartum DMI might be a potential strategy to alleviate the negative effect of NEB on performance as well as related metabolic disorders.

Approximately 3 wk before parturition, DMI starts to decline and further reduces dramatically in the last week before parturition [3-5]. On the other hand, in the last month of pregnancy the energy requirement of the dairy cow increases by about 20% to support gravid uterus growth [6]. As DMI decline in late gestation is practically unavoidable, increasing the energy density of the close-up dry period diet should help to maintain energy intake [7]. NRC (2001) recommended an approximately 5.2 MJ NE_L_/kg of DM diet for far-off dry period cows, and a 6.4 to 6.8 MJ NE_L_/kg of DM diet for close-up cows, so as to allow the rumen and its microbes to adapt to the freshly calved diet [8]. Some researchers have suggested that prepartum DMI was positively correlated with postpartum DMI and prepartum DMI should be maximized to improve postpartum performance and health [4,9]. In contrast to this viewpoint, most of the last decade’s research has shown that over consumption of energy prepartum often resulted in a slower increase in DMI postpartum compared with cows on a restricted intake [5,10,11] or fed on a low energy density diet containing wheat straw [11,12] and this was detrimental to cow health and liver function postpartum [11,13,14]. Lower DMI postpartum intensifies NEB and the mobilization of body fat results in more triacylglycerol and ketone deposition in the liver. Fatty liver has been linked to an increased incidence of metabolic disorders and poor milk yield [15]. Most of the above studies used moderate or high energy density diets with restricted DMI to control energy intake or cows were housed in a tie-stall barn, and the cows may be in a hungry condition and eager to consume more. These experimental results do not reflect the true energy intake and metabolism of transition cows housed in a free-stall barn on commercial farms, however, because transition cows normally have bunk space with free access to feed and water. Further study is needed to address this issue which is critical for dairy management.

It is very common to use moderate or even high energy density transition diets in Chinese dairy farms and the incidence of metabolic diseases is sometimes very high. However, China is short of high quality forage and abundant in low quality forage (Leymus chinensis hay, corn stover and wheat straw). Our hypothesis was that: low energy density diets containing more Leymus chinensis hay could control energy intake prepartum, having the same or better effect on EB postpartum compared to high energy density diets with restricted DMI. The objective of this study was to determine a suitable close-up dietary energy density for multiparous Holstein cows with ad libitum access to feed and housed in a free-stall barn.

## Materials and methods

### Animals and design

Animal care and use were approved and conducted in accordance with the practices outlined in the Guide for the Care and Use of Agriculture Animals in Agriculture Research and Teaching [16]. Thirty-nine New Zealand multiparous Holstein cows (average contents of milk fat, protein, and lactose were 5.49 ± 0.99%, 3.59 ± 0.38% and 4.98 ± 0.29%, respectively) were enrolled in the study. Cows were dried off at 60 d and moved into an experimental barn 35 d before expected parturition which has 48 free stalls with length × width = 230 cm × 110 cm, and the feed bunk space was 110 cm. The stocking density was about 81% (39 cows/48 free stalls). Cows were grouped according to milk production in the first 3 mo of the previous parity, body weight (BW), body condition score (BCS) and expected calving date and assigned randomly into 1 of 3 dietary treatments. All cows were enrolled in experiment at the same time. The average date before expected parturition for the 3 groups were 21.4 ± 2.5 d, 21.2 ± 2.7 d, and 21.1 ± 2.5 d when the experiment was started. The experiment was conducted from November, 2012 to February, 2013, and the average temperature and humidity during the prepartum and postpartum phases were −3.7 ± 6.3°C, −7.4 ± 4.9°C, and 46.3 ± 7.2%, 43.6 ± 6.3%, respectively.

From dry off to d 22 before expected parturition, all cows were fed the same far-off dry period diet (NE_L_5.4 MJ/kg; Table 1). From d 21 before expected day of calving until parturition, cows were blocked and assigned randomly to three groups fed a high energy density diet [HD treatment, n = 13; 6.8 MJ of net energy for lactation (NE_L_)/kg; 14.0% crude protein (CP)], or a middle energy density diet (MD treatment, n = 13; 6.2 MJ NE_L_/kg; 14.0% CP), or a low energy density diet (LD treatment, n = 13; 5.4 MJ NE_L_/kg; 14.0% CP; Table 1). The Leymus chinensis hay was the first batch of materials loaded into TMR mixer and being chopped into small particles, and then mixed with corn silage and concentrate. Different amounts of water were added to each of the three diets to adjust DM content within 51%-52%. After parturition, all the cows were provided with the same lactation diet to 70 DIM (Table 1).

**Table 1:** Composition and analysis of diets fed to Holstein cows during the dry and lactating periods

**Item**	**Far-off diet**	**Close-up diets**^**1**^			**Lactation diet**^**2**^
		**HD**	**MD**	**LD**	
Ingredients, % DM					
Leymus chinensis hay	42.8	13.3	21.7	31.3	-
Alfalfa hay	-	8.4	8.4	8.4	17.2
Whole corn silage	24.7	21.0	21.1	21.0	21.0
Corn	12.0	23.2	14.4	2.0	20.0
Extruded soybean	-	-	-	-	2.6
Soybean meal	2.1	4.5	4.0	3.7	5.8
Rapeseed meal	4.5	4.0	4.0	4.0	1.3
Cottonseed meal	5.0	4.0	4.3	4.5	1.3
Apple pomace	9.2	5.7	7.3	11.4	2.5
Whole cottonseed	-	3.3	3.3	3.3	10.4
DDGS	4.1	4.0	4.0	4.0	10.0
Palm kernel meal	5.1	3.3	3.3	3.3	-
Beet pulp meal	-	-	-	-	3.5
Calcium carbonate	1.2	1.9	1.9	1.9	1.0
Close-up premix^3^	0.1	0.4	0.4	0.4	-
Lactation premix^4^	-	-	-	-	7
Bergafat^5^	-	2.2	0.9	-	1.6
Yeast culture^6^	0.2	0.9	0.9	0.9	0.4
Sodium bicarbonate	-	-	-	-	0.9
Analysis					
DM, %	51.9	51.5	51.5	51.6	52.1
CP, % of DM	12.6	14.0	14.0	14.0	18.2
NDF, % of DM	53.3	37.4	42.9	49.8	31.7
ADF, % of DM	32.4	23.2	26.8	31.5	19.8
NFC, % of DM	22.8	35.0	30.4	23.9	36.9
Crude fat, % of DM	3.1	5.6	4.3	3.3	5.7
NE_L_, MJ/kg DM	5.4	6.8	6.2	5.4	7.2
ME, MJ/kg DM	8.7	10.7	10.0	8.8	11.1

^1^*HD* high energy density diet, *MD* middle energy density diet, *LD* low energy density diet.

^2^Lactation diet was fed to cows from parturition to 70 d in milk.

^3^Close-up premix contained (per kg of premix; DM basis): 2,200,000 IU of vitamin A, 550,000 IU of vitamin D_3_, 20,000 IU of vitamin E, 2,000 mg of vitamin PP, 3,750 mg Cu, 5,720 mg Mn, 14,850 mg Zn, 150 mg I, 180 mg Se, 120 mg Co.

^4^Lactation premix contained (per kg of premix; DM basis): 1,000,000 IU of vitamin A, 280,000 IU of vitamin D_3_, 10,000 IU of vitamin E, 1,000 mg of vitamin PP, 3,250 mg Cu, 4,800 mg Mn, 12,850 mg Zn, 140 mg I, 150 mg Se, 110 mg Co.

^5^Fractionated palm fatty acids (Berg + Schmidt, Hamburg, Germany).

^6^Diamond V XP, Diamond V Mills, Inc.

Cows were housed in a free-stall barn with delivery room, and were fed the diets as a TMR ad libitum throughout the experiment, which was offered once (at 1600 h) daily prepartum and twice (at 0730 and 1430 h) postpartum. Orts were controlled less than 8%. Cows were milked 3 times daily (at 0070, 1400, and 2030 h) in a double-48 parallel milking parlor.

### Data collections, sampling procedures, and data analysis

DMI of individual cow was determined every day from d -21 to d 35 by roughage intake control feeders (Insentec B.V., Marknesse, the Netherlands) with 48 feed bins. All cows were kept in one group and with access to different feed bins. For each visit to the bin, the system recorded the cow number, bin number, initial and final times, weights and calculated the visit duration and intake. The system’s minimum range is 0.1 kg, and it showed a high specificity (100%) and sensitivity (100%) for the feed bins and cow identification [17]. Diets were sampled weekly to determine DM contents. The particle size distribution of the TMR was determined weekly using a Penn State Particle Separator to detect sorting. Briefly, the particle size distribution of diets and orts were compared to determine if sorting occurred (≥ 15 percentage unit change on the19-mm screen) [18,19].

BW was measured at d −21, −14, −7 d relative to expected parturition and at 1, 7, 14, 21, 28, 35 DIM (WOW, XR3000, Tru-Test, New Zealand). Dry cows were weighed at 1430 h, and lactating cows were also weighed at 1430 h after milking and before diets deliver. BCS was assessed independently by two individuals on a 1 to 5 scale [20].

Cows were milked three times daily, and individual milk production except colostrum was recorded at each milking (Bou-Matic, United States). The quantity of milk was adjusted to an equal energy basis using the formula: four-percent FCM yield = (0.4 × milk yield) + [15 × (% fat/100) × milk yield]. Milk samples were obtained using milk meters at the afternoon milking on 7, 14, 21, 28, 35 and 42 DIM. Milk components (protein, fat, lactose) were determined at Beijing Dairy Cattle Center using a near-infrared reflectance spectroscopy analyzer (Seris300CombiFOSS; Foss Electric, Hillerød, Denmark).

Energy balance (EB) was calculated individually for each cow according to NRC (2001) [8]. All equations used units of megajoules per kilogram. NE_L_ intake (NEI) was determined by multiplying DMI by the mean NE_L_ density of the diet. Net energy required for maintenance (NE_M_) was calculated as BW^0.75^ × 0.08 × 120% prepartum and BW^0.75^ × 0.08 × 110% postpartum. Net energy required for pregnancy (NE_P_) was calculated as [(0.00318 × day of gestation – 0.0352) × (calf birth weight/45)]/ 0.218. Net energy required for lactation (NE_L_) was calculated as (0.0929 × fat% + 0.0563 × protein% + 0.0395 × lactose%) × milk. The equation used to calculate prepartum EB was EBPRE = NEI– (NE_M_ + NE_P_). The equation used to calculate postpartum EB was EBPOST = NEI– (NE_M_ + NE_L_).

### Statistical analysis

Five cows did not complete the study: 1 cow had a dead fetus, 3 cows had mastitis and 1 cow had a displaced abomasum. Thus, 34 cows (HD, n = 11; MD, n = 11; LD, n = 12) completed the experiment and were used in the analysis.

Individual daily DMI, NEI, NDF intake and milk yield values were condensed to weekly means before analysis; the yields and contents of milk fat, protein, lactose and EB were calculated using the weekly mean. To avoid problems with fitting covariance structure, data for DMI, NEI, NDF intake, and EB were analyzed separately for the prepartum and postpartum periods. The DMI, NEI, NDF intake, milk yield, milk components and EB were evaluated using the MIXED procedure of SPSS 16.0 (SPSS Inc.) for repeated measures with the following model: Y_ikn_ = μ + W_i_ + T_k_ + WT_ik_ + C_(ik)n_, where Y_ikn_ = an observation from the i^th^ week relative to calving, k^th^ treatment, and n^th^ cow; μ = the grand mean; W_i_ = effect of the i^th^ week; T_k_ = effect of the k^th^ treatment; WT_ik_ = effect of the week by treatment interaction; and C_(ik)n_ = random experimental error from the n^th^ cow nested within the i^th^ week and k^th^ treatment. Repeated statement was used for variables measured over time. Covariance structures including autoregressive (1), compound symmetry, and unstructured were tested. Autoregressive (1) yielded the lowest Akaike’s criterion and was finally used in our model. DMI, NEI and NDF intake for −48 h to 48 h, BW, BCS, and weekly EB were evaluated by One-Way ANOVA procedure of SPSS 16.0 (SPSS Inc.). Least squares means were computed and are presented throughout. A value of *P* < 0.05 was set as the significance level.

## Results

### DMI and NEI

The total average DMI and NEI prepartum were decreased by the reduced energy density diets (*P* < 0.05; Table 2). During the last week before parturition, the DMI of the 3 groups was not affected (*P* = 0.22) and all declined markedly, but the NEI for LD group was lower than that of HD and MD groups (*P* < 0.05). Although HD group was fed a high energy density diet, the DMI (*P* = 0.70) and NEI (*P* = 0.99) were not affected in the last 24 h before parturition. The average NDF intake was increased by the reduced energy density diets during the whole close-up period and the last week before parturition (*P* < 0.05). The total average DMI and NEI of LD group were higher than that of HD group (*P* < 0.05) during the first 5 wk of lactation, and higher in LD group compared with HD and MD groups from 4 to 5 wk of lactation (*P* < 0.05).

**Table 2:** Effect of energy density of close-up dry period diets on DMI and (NEI) in Holstein cows

**Item**	**Dietary treatment**^**1**^			**SEM**	***P*-value**		
	**HD**	**MD**	**LD**		**Diet**	**Time**	**Interaction**
DMI, kg/d							
Prepartum							
Wk −3 to −1	14.3^a^	13.6^b^	12.6^c^	0.14	<0.001	<0.001	0.87
Wk−1	12.3	11.8	11.1	0.29	0.22		
H −48 to −25	10.9	11.0	9.6	0.46	0.37		
H −24 to 0	5.0	5.6	6.3	0.61	0.7		
Postpartum							
Wk 1 to 5	18.8^b^	19.1^ab^	19.7^a^	0.13	0.04	<0.001	0.37
H 0 to 24	10.5	10.4	11.1	0.82	0.95		
H 25 to 48	11.9	12.1	12.6	0.7	0.93		
Wk 1 to 3	17.9	18.1	18.3	0.18	0.86	<0.001	0.67
Wk 4 to 5	20.1^b^	20.7^b^	21.7^a^	0.17	0.001	0.12	0.66
NEI, MJ/d							
Prepartum							
Wk −3 to −1	97.1^a^	83.3^b^	68.6^c^	0.83	<0.001	<0.001	0.42
Wk −1	83.3^a^	72.4^b^	60.2^c^	2.22	< 0.001		
H −48 to −25	73.9^a^	67.3^a^	52.0^b^	3.31	0.01		
H −24 to 0	34.1	33.9	34.3	3.64	0.99		
Postpartum							
Wk 1 to 5	136.0^b^	138.1^ab^	142.3^a^	0.95	0.03	<0.001	0.67
H 0 to 24	76.3	75.3	80.1	6.23	0.87		
H 25 to 48	86.1	97.9	91.0	5.06	0.93		
Wk 1 to 3	129.2	130.5	132.2	1.40	0.72	<0.001	0.9
Wk 4 to 5	145.2^b^	149.4^b^	156.9^a^	1.25	0.001	0.12	0.64
NDF, kg/d							
Prepartum							
Wk −3 to −1	5.3^c^	5.8^b^	6.3^a^	0.07	<0.001	<0.001	0.99
Wk −1	4.6^b^	5.1^ab^	5.5^a^	0.14	0.03		
H −48 to −25	4.1	4.7	4.8	0.20	0.32		
H −24 to 0	1.9	2.4	3.1	0.28	0.19		
Postpartum							
Wk 1 to 5	6.0^b^	6.1^ab^	6.2^a^	0.04	0.04	<0.001	0.40
H 0 to 24	3.3	3.3	3.5	0.26	0.95		
H 25 to 48	3.8	3.9	4.0	0.22	0.95		

^1^*HD* high energy density diet, *MD* middle energy density diet, *LD* low energy density diet.

^a, b, c^Values within a row with different superscripts differ significantly at *P* < 0.05.

### BCS and BW

Pretrial BCS and BW were not different among dietary treatment groups (Table 3; *P* > 0.05). The change in BCS and BW prepartum showed no significant differences among the 3 treatments (*P* > 0.05). Although all the 3 treatments gained BW prepartum, the MD group had no change in BCS and LD group lost a little BCS. The loss of BCS (*P* = 0.71) and BW (*P* = 0.55) for LD group was numerically lower than HD and MD groups during the first 5 wk of lactation.

**Table 3:** Effect of energy density of close-up dry period diets on body condition score (BCS) and BW in Holstein cows

**Item**	**Dietary treatment**^**1**^			**SEM**	***P*-value**
	**HD**	**MD**	**LD**		
BCS					
Initial BCS	3.54	3.52	3.54	0.06	0.93
Wk −3 to −1 change	0.02	0.00	−0.04	0.02	0.27
Wk 1 to 5 change	−0.42	−0.34	−0.33	0.04	0.71
BW, kg					
Initial BW	632	647	659	8.11	0.42
Wk −3 to −1 change	33.6	29.1	22.7	2.52	0.20
Wk 1 to 3 change	−32.1	−25.3	−27.4	3.25	0.73
Wk 1 to 5 change	−57.8	−49.6	−47.4	3.88	0.55

^1^*HD* high energy density diet, *MD* middle energy density diet, *LD* low energy density diet.

### Milk yield and composition

The average milk yield for MD and LD groups was higher than HD group during the first 10 wk (Table 4; *P* < 0.05). The 4% FCM yield for LD group was 2.1 kg/d less than HD group during the first 3 wk (*P* > 0.05), and was similar among the 3 groups during the first 6 wk (*P* > 0.05). The milk fat content of HD group was higher than MD and LD groups during the first 3 wk and first 6 wk (*P* < 0.05), and milk lactose content during the first 3 wk and milk lactose yield during the first 6 wk were lower than these 2 treatments (*P* < 0.05). Energy density of the diets demonstrated no effect on milk protein content, milk fat yield and protein yield during the experiment (*P* > 0.05).

**Table 4:** Effect of energy density of close-up dry period diets on milk yield and composition in Holstein cows

**Item**	**Dietary treatment**^**1**^			**SEM**	***P*-value**		
	**HD**	**MD**	**LD**		**Diet**	**Time**	**Interaction**
Milk yield, kg/d							
Wk 1 to 3	32.0	32.1	32.2	0.51	0.53	<0.001	0.78
Wk 1 to 10	36.6^b^	38.6^a^	38.7^a^	0.36	0.03	<0.001	0.99
4% FCM yield, kg/d							
Wk 1 to 3	44.0	42.4	41.9	0.78	0.55	<0.001	0.72
Wk 1 to 6	45.4	45.7	45.4	0.54	0.90	<0.001	0.91
Fat, %							
Wk 1 to 3	6.43^a^	5.87^b^	5.67^b^	0.11	0.02	0.03	0.89
Wk 1 to 6	5.96^a^	5.60^b^	5.45^b^	0.06	0.006	<0.001	0.90
Protein, %							
Wk 1 to 3	3.66	3.65	3.67	0.05	0.97	<0.001	0.54
Wk 1 to 6	3.50	3.45	3.48	0.02	0.69	<0.001	0.90
Lactose, %							
Wk 1 to 3	4.89^b^	5.07^a^	5.12^a^	0.03	0.02	0.02	0.28
Wk 1 to 6	4.99	5.10	5.12	0.04	0.06	0.002	0.12
Fat, kg/d							
Wk 1 to 3	2.05	1.94	1.90	0.04	0.33	<0.001	0.70
Wk 1 to 6	2.07	2.06	2.01	0.03	0.68	<0.001	0.89
Protein, kg/d							
Wk 1 to 3	1.17	1.20	1.21	0.03	0.57	<0.001	0.55
Wk 1 to 6	1.22	1.26	1.28	0.01	0.19	<0.001	0.94
Lactose, kg/d							
Wk 1 to 3	1.58	1.69	1.72	0.03	0.12	<0.001	0.69
Wk 1 to 6	1.77^b^	1.90^a^	1.91^a^	0.02	0.04	<0.001	0.99

^1^*HD* high energy density diet, *MD* middle energy density diet, *LD* low energy density diet.

^a, b^Values within a row with different superscripts differ significantly at *P* < 0.05.

### Energy balance

In the close-up dry period, there was significant difference among the 3 treatments in EB (Table 5; *P* < 0.001). HD, MD and LD groups consumed 30.1, 15.9 and 1.3 MJ/d more than the prepartum NE_L_ requirement, respectively; and achieved 149.8%, 126.2% and 101.1% of their calculated energy requirement, respectively. During the last week before calving, LD group consumed 87.4% of their energy requirement and the other 2 treatments both exceeded 100%. LD group had a milder NEB compared to the other 2 treatments during the first week of lactation. During the first 4 wk postpartum, HD, MD and LD groups achieved 72.7%, 73.1% and 75.2% of their calculated energy requirement, respectively. In all treatments, the most marked NEB appeared in approximately the third and fourth week.

**Table 5:** Effect of energy density of close-up dry period diets on EB in Holstein cows

**Item**	**Dietary treatment**^**1**^			**SEM**	***P*-value**		
	**HD**	**MD**	**LD**		**Diet**	**Time**	**Interaction**
EB, MJ/day							
Prepartum							
Wk −3 to −1	30.1^a^	15.9^b^	1.3^c^	0.94	< 0.001	< 0.001	0.34
Wk −3	42.3^a^	26.8^b^	7.5^c^	3.22	< 0.001		
Wk −2	37.2^a^	20.9^b^	3.3^c^	2.93	< 0.001		
Wk −1	15.9^a^	6.7^b^	−8.4^c^	2.34	< 0.001		
Postpartum							
Wk 1 to 4	−45.2	−46.0	−43.1	2.2	0.66	0.09	0.99
Wk 1	−43.5	−39.7	−31.0	4.27	0.49		
Wk 2	−41.0	−40.2	−38.9	4.06	0.98		
Wk 3	−51.9	−53.1	−52.3	4.48	0.99		
Wk 4	−47.3	−47.7	−43.9	3.05	0.86		
EB, % of requirement							
Prepartum							
Wk −3 to −1	149.8	126.2	101.1				
Wk −1	125.6	109.7	87.4				
Postpartum							
Wk 1 to 4	72.7	73.1	75.2				
Wk 1	72.7	73.5	79.2				

^1^*HD* high energy density diet, *MD* middle energy density diet, *LD*, low energy density diet.

^a, b, c^Values within a row with different superscripts differ significantly at *P* < 0.05.

## Discussion

### DMI and NEI

The average DMI for the 3 treatments was 12.6-14.3 kg/d prepartum, which was higher than our previous study with cows in a tie-stall barn (12.3 kg/d in cool autumn and 8.0 kg/d in hot summer) [21]. In Janovick’s study [12], the cows housed in tie stalls and fed the same high NE_L_ diet (6.8 MJ/kg) had less DMI (13.1 v. 14.3 kg/d) compared with our study, but those fed a similar low NE_L_ diet (5.2 v. 5.4 MJ/kg) had a slightly higher DMI (12.8 v. 12.6 kg/d), and the BW of our experimental cows were much lower (approximately 650 v. 730 kg). Leymus chinensis hay has high NDF (68% ~ 74%, DM) and ADF (35% ~ 40%, DM) contents, and it’s effective ruminal NDF degradability is 26% ~ 31%, and is abundant in the Northeast of China. In the current study, cows fed a low energy density diet containing much Leymus chinensis hay had a significantly lower DMI prepartum. In the last 48 h before calving, however, the DMI for the 3 treatments had no significant difference. The results revealed that a high NDF content diet gave a small change in DMI prepartum. Other studies have shown that diets supplemented with much wheat straw had lower DMI and DMI decline rate prepartum [11,22,23]. The average proportion of the19-mm screen for diets of HD, MD, LD groups were 23.2%, 20.4% and 14.7% as fed basis prepartum, respectively; and they were 35.1%, 29.8% and 19.3% for orts of HD, MD, LD groups, respectively. All the changes on the19-mm screen were less than 15 percentage unit, indicating no sorting has occurred.

58.6-60.7 MJ/d is recommended by NRC (2001) for NEI of close-up dairy cows [8]. In our study, the average prepartum NEI was 97.1, 83.3 and 68.6 MJ/d for HD, MD and LD, respectively, which were higher than the energy requirements recommended by NRC (2001) [8]. Other studies have also confirmed that close-up cows fed with moderate or low energy density diets would easily consume more energy relative to their requirements if DMI is not limited [11,24]. But the energy consumption of LD group fed a higher level of Leymus chinensis hay was closest to the NRC (2001) recommendation [8]. Diets high in wheat straw in other studies have also shown the effect of controlling energy intake prepartum [11,12]. The LD group had significantly higher NDF intake compared with HD group during the last week before parturition. In the last 24 h before calving, the NDF intake of LD group increased 63% compared with HD group, but the NEI was similar among the 3 treatment groups. Therefore we could conclude that DMI in the last 24 h before calving was not decided by rumen volume, but rather by energy requirement.

After parturition, LD group had higher DMI compared with HD and MD groups. These results concur with another two studies showing that large changes in DMI prepartum have been related to lower DMI postpartum [25,26]. This effect can be explained as follows, 1) the dietary palatability changed more for the LD group from dry cow diet to lactation diet, and the average DMI of the first and second 24 h periods after calving were 0.6 kg and 0.7 kg higher than HD group in this study. 2) Small changes of DMI prepartum may lead to less metabolic disorders which could decrease DMI. Some studies have demonstrated that overfeeding energy prepartum results in a greater number of health problems postpartum [11,24,27].

### Lactation performance

The reduced energy density of close-up diets increased the milk yield, but did not significantly affect 4% FCM yield. During the first 3 wk, average milk yields of the 3 treatments were similar, but the 4% FCM yield was 2.1 kg/d more for HD group than LD group due to a significantly higher milk fat content. Douglas et al. [24] also showed that cows on restricted intake diets prepartum had a numerically higher milk yield (1.5-2.5 kg/d) compared to those on ad libitum intake. On the contrary, cows fed a 150% of requirement energy diet prepartum tended to produce more milk in wk 1 to 8 than cows fed a 100% and 80% requirement energy diets [12]. In the above two studies, milk fat content for cows with more energy consumption prepartum was significantly or numerically higher than lower energy consumption cows which was in agreement with our study. Janovick et al. [12] reported that high milk fat content might be due to a greater mobilization of body stores in the early lactation period. Blood nonesterified fatty acids (NEFA) could account for as much as 40% of milk fat content on 4 DIM [1]. In this study, the plasma NEFA concentration for HD group was significantly higher than that of LD group during the first 2 wk of lactation (605.7 v. 464.8 μEq/L, the data were not published), and the numerically higher BW and BCS loss of HD group could be explained by more mobilization of adipose tissue, which resulted in a high milk fat content. Although milk lactose content is stable, the milk lactose content for HD group was significantly lower than MD and LD groups during the first 3 wk in the current study. A similar result was reported by Janovick et al. [12]. However, this result was not observed by some others [11,28].

### Energy balance

Douglas et al. [24] reported that cows fed 6.3 MJ/kg NE_L_ diet in the whole 60 d dry period consumed an average of 159% of the NRC (2001) requirements for NE_L_. In the current study, cows on moderate or even low energy diets offered ad libitum intake during the close-up period consumed more energy relative to their requirements. The HD group was almost in positive EB in the whole close-up period, but the NEI was markedly lower than requirements due to a sharp decrease in DMI in the last 3 d before parturation. A 5.4 MJ of NE_L_/kg diet could meet the energy requirements of cows before d 12, and the NEB became much more serious with the onset of calving. All the 3 treatment cows gained BW prepartum, but LD group lost BCS. Janovick et al. [12] also reported that dry cows offered a diet meeting 80% of energy requirements lost BCS but gained18 kg over the dry period. We could conclude that the increase of BW was attributable to the rapid growth of the fetus and placenta in late pregnancy, and not to maternal BW increase.

Although the average postpartum EB was not significantly affected by prepartum diets, HD group had 3.8 and 12.5 MJ/d more energy deficiency than MD and LD groups during the first week of lactation, respectively. Other studies have also showed that overfed cows in the dry period were in more NEB postpartum [11,12]. The higher milk fat content for HD group during the first 3 wk of lactation also implied a more marked NEB in the current study.

## Conclusions

Feeding a high energy density diet prepartum resulted in large decline in DMI prepartum, higher milk fat content and lower milk lactose content during the first 3 wk of lactation. The low energy density diet was beneficial in controlling NEI prepartum, increasing milk yield and DMI, and alleviating NEB postpartum.

## Abbreviations

DMI: Dry matter intake; EB: Energy balance; NE_L_: Net energy for lactation; HD: High energy density diet containing 6.8 MJ NE_L_ /kg; MD: Middle energy density diet containing 6.2 MJ NE_L_ /kg; LD: Low energy density diet contaning 5.4 MJ NE_L_ /kg; DIM: Day in milk; DM: Dry matter; NEB: Negative energy balance; BW: Body weight; BCS: Body condition score; CP: Crude protein; NDF: Neutral detergent fibre; ADF: Acid detergent fibre; NEI: NE_L_ intake; NE_M_: Net energy required for maintenance; NE_P_: Net energy required for pregnancy; EBPRE: Prepartum EB; EBPOST: Postpartum EB.

## Competing interests

The authors declare that they have no competing interests.

## Authors’ contributions

WH, YT, AS, AY and ZW carried out the experiments. SL, ZC and YW participated in the design of the study, and drafted the manuscript. All authors read and approved the final manuscript.
